# Interacting effects of sex and age on immune responses in a polygynous bat with male-biased mortality

**DOI:** 10.64898/2026.02.27.708485

**Published:** 2026-02-27

**Authors:** Jack G. Rayner, Danielle M. Adams, Najib M. El-Sayed, David M. Mosser, Gerald S. Wilkinson

**Affiliations:** 1. Department of Biology, University of Maryland, College Park, MD 20742; 2. Department of Cell Biology and Molecular Genetics, University of Maryland, College Park, MD 20742; 3. Center for Bioinformatics and Computational Biology, University of Maryland, College Park, MD 20742

## Abstract

Bats have attracted considerable recent interest for their extraordinary longevity and ability to withstand infection by a range of pathogens without major harm. However, few studies have examined immune responses as a function of age, sex, or social status. We investigated sources of individual immune variation by comparing whole-blood transcriptomes of wild greater spear-nosed bats, *Phyllostomus hastatus*, before and after ex vivo exposure to lipopolysaccharide, a membrane component of gram-negative bacteria. This species exhibits an extreme harem-polygynous mating system, which has pervasive consequences on life history traits, including male-biased mortality. We observe clear differences in immune responses, with males and older bats each mounting stronger inflammatory responses. Males also show steeper patterns of age-related variation in immune profiles, suggesting their earlier mortality is associated with accelerated immunosenescence. We did not detect largescale differences in immune responses between males of different social status, suggesting that—unlike in many primates—immune variation is not strongly influenced by social adversity. Our findings support recent calls for a more nuanced approach to understanding immune adaptations in bats that considers the diverse ways in which species and individuals differ in ecology, resource allocation, and selection.

## Introduction

Animals of the same species frequently differ markedly in their responses to immune challenges (1). Much of this variation likely arises because individuals differ in life history and thus resource allocation (2), with immune investment associated with both direct and indirect costs (e.g., due to resource allocation and damaging long-term consequences, respectively) (3). Despite this, individual differences are often overlooked in studies that seek to identify genomic and molecular characteristics that underpin increased immune efficiency and extended longevity, which often involve comparisons between distantly related taxa. Research that exploits variation between individuals of the same species has the opportunity to generate important insights into selection pressures and trade-offs underlying individual differences in immunity, health, and longevity at both micro- and macroevolutionary scales (2).

Bats have received intense recent scrutiny in fields of gerontology and immunology (4, 5), owing to their extraordinary longevity (6, 7) and ability to withstand infection from highly virulent pathogens, such as ebolaviruses (8) and MERS-CoV (9), often without visible pathology. This ability has been shaped by distinctive features of their ecology and life history. Perhaps most strikingly, many bats form high density colonies that promote pathogen transmission (5, 10), providing ample opportunity for genetic adaptation to threats posed by virulent diseases. A common view is that this gregariousness generated selection for bats to ‘tolerate’ high frequencies of infection without mounting severe immune responses that exert harmful long-term consequences (5, 11), e.g., via dampened inflammasome activation (12–14). On the other hand, some studies of bats have observed constitutive elevated expression of innate immune effectors, such as type I interferons (15–17), that appear to confer resistance in the early stages of viral infection (16, 17). Besides their gregariousness, bats are also unique among mammals in being capable of powered flight. Flight is extremely metabolically costly (18) and is thought to lead to elevated levels of DNA damage, which is proposed to have selected for reduced immune sensitivity to cytosolic DNA, otherwise symptomatic of viral infection (19). This view is supported by observations of dampened STING-dependent interferon activation (20) and TLR-9 mediated DNA-sensing (21) in some bat species. Additionally, elevated body temperature during flight (18, 22–24) might evoke a fever-like response that aids in pathogen control (25) or modulate antibody sensitivity (26), though methodological confounds in measuring bat body temperatures have been argued to challenge the validity of these hypotheses (27).

A criticism recently levelled at studies of bat immunological adaptations is that they often overlook important interspecific variation, despite differences in ecology and pathogen exposure (28). It is therefore not surprising that intraspecific variation in immune responses has also been overlooked, in part due to approaches capitalising on techniques in comparative genomics (29–31) and functional assays (14, 20, 21) that largely ignore individual differences. Yet, studies of other taxa have observed this variation can be substantial (32). In particular, immune responses frequently differ markedly between the sexes (33–35), and individuals of different age (36, 37) or social status (38, 39). Besides direct genetic influences (35, 40), much of this variation in immune function likely arises via differences in life history (41). For example, males of many species, including bats, are subject to strong competition for the opportunity to mate with females (42, 43). Males therefore tend to invest heavily in the development and maintenance of costly secondary sexual traits, such as large body size, weapons or ornamentation. These traits can increase male reproductive fitness but likely involve reallocation of resources from other traits involved in somatic maintenance (44), potentially including immune function (2, 3), ultimately contributing to sex-biased mortality (45, 46).

We investigated variation in immune responses in wild greater spear-nosed bats, *Phyllostomus hastatus*. This species is remarkable for its extremely polygynous mating system, in which dominant ‘harem’ males aggressively defend groups of approximately 15 to 25 females, whereas subordinate ‘bachelor’ males congregate in more diffuse assemblages (47). This competitive mating system shapes inter- and intrasexual variation in multiple life history traits. Harem males must defend groups of females and exhibit elevated cortisol levels throughout the year (48). Moreover, males—especially bachelors—experience accelerated mortality, with median and maximum lifespans about half those of females (49). In the current study, we examined differences in transcriptomic responses to lipopolysaccharide (LPS): a pathogen-associated molecular pattern (PAMP) present in the cell walls of gram-negative bacteria. LPS induces potent pro-inflammatory immune responses in mammals that must be properly regulated to avoid damaging consequences, such as septic shock (50). We first verified that exposure to LPS stimulated the expected immune response, testing for effects on the expression of genes and pathways involved in innate immunity as well as those that respond to LPS in other mammal species. We then assessed differences in immune responses via comparisons between immune stimulated transcriptomes. We predicted that stressful consequences of extreme polygyny in *P. hastatus* would be associated with differences in immune responses between sexes and males of different social statuses; and that immune responses would also vary between bats in relation to age, potentially reflecting immunosenescence. Given prior observations of male-biased mortality and epigenetic aging in this species (49), we further predicted steeper age-associated changes in immunity among males. Our tests of these predictions will have important consequences for understanding the forces that shape immunological variation in wild populations, and offer insight into the factors that contribute to immune efficiency and extended longevity in bats.

## Results

We sequenced transcriptomes of whole-blood samples collected from wild *P. hastatus* at two locations in Trinidad, which we either immediately preserved (‘untreated’, N = 70), or incubated with lipopolysaccharide for three hours prior to preservation (‘LPS-treated’, N = 96) to simulate infection and provoke an immune response. Unless individual ages were known from mark-recapture records, we also collected a 4mm wing skin tissue biopsy, from which we extracted DNA for methylation profiling to estimate chronological age based on a previously published methylation clock (51), which accurately predicts chronological ages of male and female *P. hastatus* (49). We balanced whole-blood transcriptome sequencing across males and females, and included the full range of adult ages in each sex (Fig. S1): this precludes balancing age ranges across sexes, due to pronounced sex-differences in mortality (49). For a parallel study (Wilkinson et al. in review), we also created a blood smear to estimate the neutrophil-to-lymphocyte ratio, commonly used as a biomarker of inflammation, which was consistently male-biased and increased with age in both sexes. Where stated, we include this measure as an additional covariate in models of differential gene expression, to investigate the influence of individual variation in leukocyte composition.

### LPS elicits a strong pro-inflammatory response in bats

**Figure 1.**
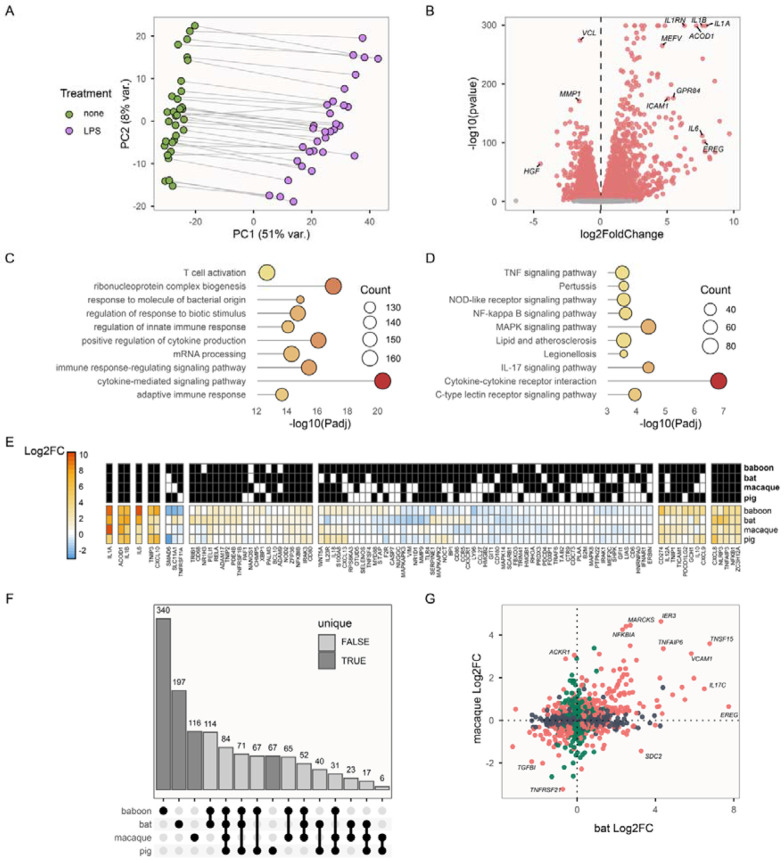
LPS affects expression of genes involved in proinflammatory immune responses **A)** PCA of paired untreated and LPS-treated blood samples in bats. ** B)** Volcano plot with differentially expressed genes highlighted in red. X-axis values greater than 0 indicate up-regulated expression in LPS-treated blood. **C)** The top 10 overrepresented biological process gene ontology categories, and **D)** KEGG pathways among up-regulated genes. Point size indicates the number of DE genes in each GO category, or the number of genes in each KEGG pathway, and points are coloured according to X-axis values. **E)** Heatmap visualising log_2_ fold-changes following LPS treatment of genes in the ‘response to LPS’ gene ontology category, in four mammal species. Genes are hierarchically clustered by expression similarity. Black and white tiles indicate whether each gene was significantly differentially expressed (black) in the respective species. **F)** An ‘upset’ plot showing numbers of genes uniquely up-regulated following LPS treatment in each species (dark grey) and that are up-regulated in more than one species (light grey bars). **G)** Log_2_ fold-changes following LPS exposure in bats and macaques. Dark grey points are genes that responded significantly to LPS in bats only, green points indicate genes that responded significantly to LPS in macaques only, and peach-colored points are those that responded significantly in both species, in either direction.

Treatment of blood samples with LPS *ex vivo* provoked a strong immune response, highlighting core genes and pathways associated with inflammation. In a set of paired untreated and LPS-treated blood samples from 32 bats, 16 of each sex, principal component analysis confirmed that LPS treatment explained substantial transcriptomic variation: untreated and LPS-treated samples were separated on the first principal component (explaining 51% of variation among the top 1000 variable genes; Fig. 1A). The second principal component explained 8% of variation and was associated with interindividual variation (Fig. 1A). A paired differential gene expression analysis revealed LPS treatment significantly affected expression of 7,394 genes (of 16,046 included in the analysis), with many important immune-associated genes up-regulated following LPS treatment (Fig. 1B). These up-regulated genes were overrepresented for immune-related biological processes including ‘response to molecule of bacterial origin’, ‘regulation of innate immune response and ‘positive regulation of cytokine production’ (Fig. 1C), and were also overrepresented in key immunological and inflammatory KEGG pathways, including TNF, NF-kappa β, cytokine-cytokine receptor and IL-17 signalling (all P_adj._ < 0.05) (Fig. 1D). For example, specific inflammation-associated transcription factors such as *NFKB1* (log_2_ fold-change: 2.54) and cytokines such as *IL1A* (log_2_FC: 7.85), *IL1B* (log_2_FC: 7.61) and *IL6* (log_2_FC: 7.60), were all strongly up-regulated (P_adj._ < 1.06e-100) following LPS stimulus.

To further assess whether LPS treatment stimulated the expected immune response, we compared transcriptomic responses to LPS in whole blood samples of *P. hastatus* with those of peripheral blood mononuclear cells (PBMCs) collected from rhesus macaques (*Macaca mulatta*) (38) and pigs (*Sus domesticus*) (52), and leukocytes of yellow baboons (*Papio cynocephalus*) (39). We initially visualised log_2_ fold-changes across 124 gene symbols that are annotated for involvement in the ‘response to lipopolysaccharide’ gene ontology category (GO:0032496) in humans and that significantly responded to LPS in at least one of the four species, observing that responses to LPS appeared largely conserved (Fig. 1E). Of a few notable exceptions, *IL23R* was significantly down-regulated in bats following LPS stimulus but significantly up-regulated in each of the other three mammals, whereas *TNFRSF11A* and *SLC11A1* were each significantly up-regulated following LPS stimulus in bats but significantly or non-significantly down-regulated in each of the other three mammals (Fig 1E). We subsequently directly compared responses to LPS across 2,600 single-copy orthologous genes retained in the differential expression analysis of all four species (Fig. 1F–G). Of 598 such genes significantly up-regulated in *P. hastatus* following LPS treatment, 401 (67%) were also significantly up-regulated in at least one other species: 3.85 times as many as expected by chance (Fisher’s exact test: P < 0.001). These were overrepresented for a host of immunity-related biological processes (Fig. S2), whereas genes that were uniquely up-regulated in *P. hastatus* were not overrepresented in any category. In sum, considering differences in methodology between studies, the responses we observed to LPS in *P. hastatus* appear largely consistent with those observed in other mammals.

Although transcriptional responses to LPS resemble those of other mammals, RNAs could be subject to post-transcriptional regulation that mediates this response, such as via micro-RNAs. We sequenced small RNAs from 40 paired samples of *P. hastatus*, 20 untreated and 20 LPS-treated, and found that the abundance of 60 putative miRNAs (of 355 included in the analysis) was affected by LPS-treatment, 31 of which were up-regulated (Fig. S3). The 27 human-annotated miRNAs that responded to LPS treatment were over-represented for putative targets in biological processes including ‘viral process’ (FDR = 8.09e-35), ‘positive regulation of I-kappaB kinase/NF-kappaB signaling’ (FDR = 3.71e-06) and ‘interleukin-1-mediated signaling pathway’ (FDR = 1.93e-05), and in the KEGG pathway ‘bacterial invasion of epithelial cells’ (FDR = 7.24e-8). miRNA profiles of LPS treated samples therefore also appeared broadly consistent with expectations following an immune stimulus.

### Immune responses differ markedly by sex and age

**Figure 2.**
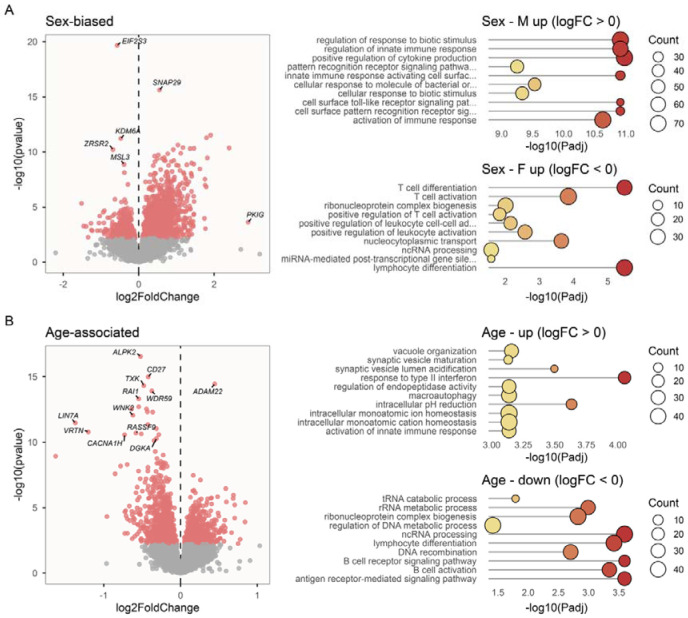
Sex and age-related differences in immune-stimulated transcriptomes. **A)** Volcano plot showing results from differential expression analyses for sex-biased genes, with significantly differentially expressed genes highlighted in red. Note: three very strongly sex-biased genes (P_adj._ < 1e-86) including *Xist* and *ZFX*, that were similarly sex-biased in untreated samples, are omitted from the plot. Panels to the right show gene ontology categories overrepresented among male- and female-biased genes. **B)** Volcano plot showing results from differential expression analyses for age-associated genes, with significantly differentially expressed genes highlighted in red. Panels to the right show gene ontology categories overrepresented among up- and down-regulated genes.

We predicted that immune responses would differ between sexes and ages, due to differences in life history and senescence. In support of this, large numbers of genes were differentially expressed between sexes (N = 1,827) and ages (N = 1,682) (Fig. 2) across 96 LPS-treated blood transcriptomes. This is considerably more than we observed across 70 untreated blood samples, in which just 136 genes were sex-biased and 29 genes exhibited age-associated variation. Substantial transcriptomic differences between sexes and ages among LPS-treated, but not untreated, blood samples therefore appear to reflect variation in immune response. Consistent with this interpretation, LPS-sensitive genes (those significantly up-regulated in blood samples following LPS-treatment, see above) were strongly overrepresented among genes showing sex and age-associated expression differences in immune-stimulated transcriptomes (Fisher’s exact tests; sex-biased genes: odds ratio = 2.815, P < 0.001; age-associated genes: odds ratio = 1.505, P < 0.001). The 527 genes showing female-biased expression in immune-stimulated samples were overrepresented for genes involved in biological processes associated with adaptive immunity, such as lymphocyte differentiation and activation, whereas the 1,300 genes showing male-biased expression were over-represented for biological processes involved in innate immune responses, such as cytokine production and Toll-like receptor signalling (Fig. 2A). Similarly, the 771 genes that increased in expression with age were overrepresented for genes involved in activation of the innate immune response, whereas the 911 genes that declined with age were overrepresented for adaptive immunity-related processes, such as lymphocyte differentiation and antigen receptor-mediated signalling (Fig. 2B). We did not observe similar sex and age-associated variation in miRNA abundance in either untreated or LPS-treated blood (Supplemental Information; Fig. S4).

Blood leukocyte composition influenced sex and age-related differences in immune-stimulated transcriptomes. Inclusion of log-transformed neutrophil-to-lymphocyte ratio (NLR) as a covariate in models of gene expression greatly reduced numbers of sex-biased and age-associated genes among all LPS-treated transcriptomes (from 1,827 to 97 sex-biased, and from 1,682 to 621 age-associated, genes), with NLR itself significantly associated with the expression of 5,779 genes. This suggests that gene expression profiles in LPS-treated samples are strongly influenced by differences across sexes and ages in blood cell composition. By contrast, inclusion of NLR had little to no effect on the magnitude of differential gene expression in untreated blood samples (from 136 to 154 sex-biased, and 29 to 18 age-associated, genes), despite being associated with differential expression of 4,454 genes.

### Immunological variation is weakly associated with social status

By contrast with the substantial sex and age-related variation in immune profiles, relatively few (N = 41) genes differed in expression between LPS-stimulated transcriptomes from 31 bachelor and 17 harem-controlling males, while accounting for status-associated differences in age, as harem-controlling males tend to be older (48). These status-associated genes were not significantly overrepresented in any gene ontology categories but were enriched for genes up-regulated following LPS treatment (odds ratio = 3.485, P < 0.001). Notably, this enrichment of LPS-responsive genes was especially strong among the 22 genes that were up-regulated in harem males (odds ratio = 11.693, P < 0.001), potentially consistent with a stronger immune response in dominant harem males, at least among this limited subset of status-associated genes. (Fig. S5)

### Males respond more strongly to immune stimulus

Male and female bats differed in their responses to LPS, but whether these differences represent immune responses of differing intensities is not immediately clear. Two initial lines of enquiry supported the view that males responded more strongly to LPS. First, among paired untreated and LPS-treated samples, males tended to exhibit larger responses to LPS across LPS-sensitive genes identified in the analysis above. Sex-specific log_2_ fold-changes across these LPS-sensitive genes were very strongly correlated (Pearson’s r = 0.961, df = 7392, P < 0.001), but those that changed in the same direction in both sexes (the vast majority: 99.7%) exhibited a consistent male-bias in log_2_-fold change (paired t-test: t = 8.074, df = 7367, P < 0.001), with a mean difference of 0.028 (95% CI: 0.021, 0.035).

**Figure 3.**
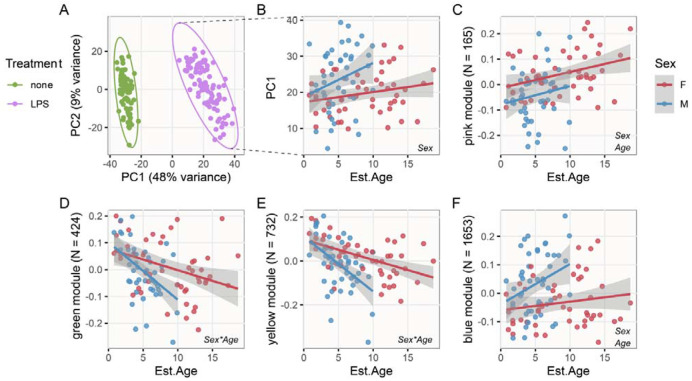
Interacting effects of sex and age on transcriptomic immune profiles. **A)** The results of principal component analysis based on the 1000 most variable genes, separating untreated and LPS-treated samples on PC1, with ellipses delineating groups of samples at 0.99 confidence level. Panel **B)** illustrates sex and age-related differences in PC1 values in LPS-treated samples. Panels **C-F**) show patterns of expression for representative eigengenes from gene expression modules obtained by WGCNA. Text annotations indicate significant predictor variables from the linear mixed model reported in the main text. In all panels, lines represent results of sex-specific linear regression and 95% confidence intervals.

Next, we investigated sex and age-related differences across the full dataset of 70 untreated and 96 LPS-treated transcriptomes, and found that male LPS-treated transcriptomes diverged more strongly from those of untreated transcriptomes. For this analysis, we collapsed gene expression variation across the top 1000 most variable genes using principal component analysis. The first principal component (PC1) separated untreated and LPS-treated transcriptomes (Fig. 3A), so we treated PC1 values in LPS-treated samples as a proxy for the magnitude of response to LPS treatment (38). A linear mixed model incorporating sequencing batch as a random intercept supported the interpretation, above, that males responded more intensely to LPS stimulus (Sex: Wald’s X^2^_1_ = 10.198, P = 0.001) and also indicated responses to LPS tended to be stronger among older bats (Age: X^2^_1_ = 3.639, P = 0.056), but did not support a sex by age interaction despite a visual trend towards a steeper age slope in males (X^2^_1_ = 1.457, P = 0.227; term not included in final model) (Fig. 3B). Sex and age-related variation in PC1 values dissipated (P > 0.50) when log-transformed neutrophil-to-lymphocyte ratio was included in the model as a covariate (X^2^_1_ = 51.211, P < 0.001), supporting the previous inference that variation in immune response is strongly influenced by differential leukocyte abundance.

### Males exhibit accelerated immunosenescence

To investigate immune variation associated with sex and age at a finer scale while accounting for covariance between genes, we next implemented signed, weighted gene co-expression network analysis (WGCNA) across LPS-treated transcriptomes. This analysis identified four gene expression modules, containing between 165 and 1,653 genes, for which representative ‘eigengenes’ were associated with sex and/or age (t-test: Student’s asymptotic P_adj__._ < 0.05). Among these, a ‘pink’ module was more highly expressed in older bats (linear regression: X^2^_1_ = 6.651, P = 0.001) and in females (X^2^_1_ = 8.066, P = 0.005) but showed no evidence of a sex by age interaction (Fig. 3C). Two of the other three modules reported significant sex by age interactions (‘green’: X^2^_1_ = 4.11, P = 0.043; ‘yellow’: X^2^_1_ = 6.350, P = 0.012), with the remaining ‘blue’ module also indicating some support for a sex by age interaction (X^2^_1_ = 2.763, P = 0.096). In each case, males exhibited steeper slopes of age-related variation (Fig. 3D–F). All four of these sex or age-associated modules were overrepresented for genes involved in immune-related biological processes (Fig. S7): of particular interest, ‘green’ and ‘yellow’ modules, among which expression shows male-biased age-related declines, were over-represented for terms related to B and T cell activation, whereas the ‘blue’ module was overrepresented for genes related to innate immunity and inflammation and showed some evidence of male-biased age-associated increases in expression.

**Figure 4.**
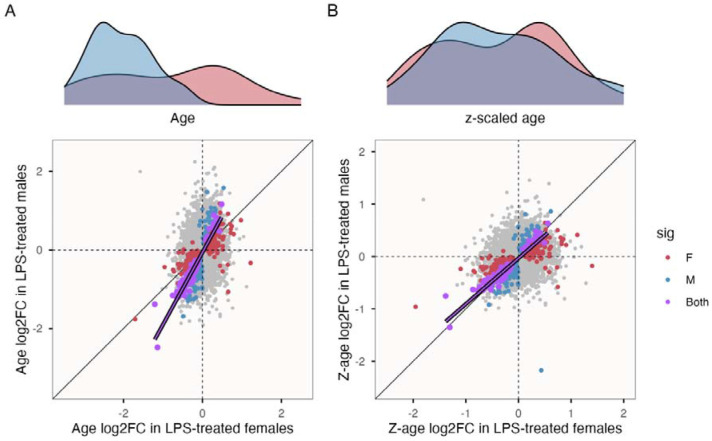
Male-biased slopes of age-associated variation in immune-treated samples. **A)** Sex-specific age distributions. **B)** Sex-specific sample age distributions when age is z-scaled independently for each sex. Corresponding plots below compare slopes of age-related variation in gene expression between sexes in immune-treated samples. Red and blue points show genes that were significantly age-associated females and males, respectively, with purple points those significant in both sexes. The purple line shows the result of linear regression across genes that are significantly age-associated in both sexes.

Male-biased slopes of age-related variation in immune-stimulated transcriptomes were reiterated by analyses at the gene level. The large majority of age-related differences showed consistent sign across sexes; of 682 genes that were significantly age-associated in either sex, 664 (97.36%) showed the same direction of change in males and females (Fisher’s exact test: odds ratio = 17.14, P < 0.001). All 120 genes identified as significantly age-associated in both sexes showed concordant directions of change, and the average male slope was 1.35 ± 0.03 SE times that of females (paired t-test: t = 22.327, df = 119, P < 0.0001) (Fig. 4A). A large majority of these consistently age-associated genes (N = 107) were negative correlated with age, and were strongly overrepresented for adaptive immunity-related biological processes (Fig. S8).

The above results support a pattern of stronger age-related immune variation in males. This pattern could be related to their elevated mortality rate. If this were the case, then old males and females might show similar immune profiles once their different lifespans are taken into account. To explore this, we reran the above analyses after performing sex-specific z-scaling of ages: in which male and female ages are each quantified in terms of standard deviations from the mean age in the respective sex (Fig. 4B cf. Fig. 4A). The interpretation of male-biased immunosenescence subsequently dissipated: first, of the 120 genes identified as age-associated in the same direction in both sexes, none differed in slope after sex-specific scaling (paired t-test: t = 0.363, df = 119, P = 0.717). Similarly, sex-specific scaling of ages removed evidence of sex-by-age interactions among co-expressed gene modules (all P > 0.366, Fig. S9). Finally, sex-specific scaling also reduced the number of sex-biased genes among LPS-stimulated samples, from 1,827 to 1,089. In sum, these findings suggest that male-biased patterns of age-related variation in part reflect sex differences in mortality and thus age distribution.

## Discussion

How bats regulate immune responses under frequent exposure to a range of pathogens has attracted considerable interest, but studies have rarely assessed differences between individuals of the same species. This is despite widespread appreciation of individual-level differences in immunity in animals (53), and considerable variation in ecology and life history between individuals of different sexes and social status across a taxonomically diverse range of bat species (42, 43, 54, 55). As predicted, we observed strong sex and age-related differences in immune responses of *P. hastatus*, emphasising the importance of appreciating sources of intraspecific variation. In particular, males—which live approximately half as long as females—responded more strongly to an immune stimulus, up-regulating genes involved in innate immunity and inflammation. Genes involved in innate immunity were also up-regulated in older bats, whereas genes associated with adaptive forms of immunity were down-regulated, fitting expectations under immunosenescence (37). Unexpectedly, we observed little evidence of immune variation between males of contrasting social status, a finding which deviates from studies in other mammals, particularly primates (38, 39).

It has been predicted that male vertebrates should tend to invest more in innate immune defences and inflammation than females (56), as their fitness is less strongly impacted by long-term physiological costs. Our findings are consistent with this, but we also observed a pronounced shift towards innate over adaptive forms of immunity in older bats, with slopes of age-associated expression variation notably steeper in males. This suggests that sex differences in immune responses are influenced at least in part by male-biased rates of immunosenescence. This aligns with broader patterns of male-biased actuarial and physiological senescence: prior research in *P. hastatus* has documented sex differences in age-related variation across hormone profiles (48), DNA methylation (49), telomere length (57), and neutrophil-lymphocyte ratios (Wilkinson et al., in review). The latter is especially pertinent to the current study, as we found immune variation was strongly influenced by neutrophil-to-lymphocyte ratio, accounting for much of the sex and age-related variation we observe in immune-stimulated samples. A caveat of the interpretation of male-biased immunosenescence is that our data are cross-sectional, so sex-differences in age-related slopes could feasibly be influenced by sex differences in selective disappearance. However, accounting for sex differences in age distribution led to the disappearance of this pattern, providing circumstantial support for the view that differences in age-related variation relate to male-biased mortality. In addition, cross-sectional sex and age-related differences in neutrophil-to-lymphocyte ratio, which strongly influenced immune profiles, are reflected in longitudinal comparisons (Wilkinson et al. in review). Finally, male-biased selective disappearance might be expected to disguise rather than accentuate patterns of immunosenescence. In sum, our results indicate a pronounced trend towards male-biased immunosenescence in *P. hastatus*, likely influenced by the stressful polygynous mating system.

Skewed mating systems generate differences in social status, which are expected to contribute to immune variation (53). Studies of humans and other primates have found social adversity is associated with stronger proinflammatory responses (38, 58–60), frequently associated with aging phenotypes (61). However, the nature of this relationship is variable across taxa, even among primates, with high-status male baboons (*Papio cynocephalus*) exhibiting up-regulation of genes involved in innate immunity and inflammation (39), and accelerated epigenetic aging (62). Additionally, lower status individuals of a variety of species display better health (53) and reduced parasite loads (63). In *P. hastatus*, bachelor and harem-controlling males differ across a range of life history traits (47–49), suggesting ample opportunity for social status to influence male immune profiles. Yet, we did not observe strong differences in responses to immune stimulus between these male groups. This could challenge the taxonomic generality of the view that social status has important consequences for immunity (64), at least outside of primates and other taxa with complex social hierarchies. However, male reproductive status is just one facet of social variation in *P. hastatus* populations (65). Future work also incorporating, for example, female differences in social connectivity and status could offer a more comprehensive evaluation of social differences in immunity in this species.

Bats have been proposed to benefit from attenuated immune responses, particularly with respect to inflammation, potentially contributing to their extraordinary longevity (5). We observed a pronounced albeit variable proinflammatory response to LPS in *P. hastatus*, which outlive most similarly sized mammals. This is not altogether surprising, as innate immunity and inflammatory signalling are evolutionarily conserved (66), but is nevertheless noteworthy in the context of reports of dampened inflammatory responses in multiple bat species. For example, bone-marrow-derived macrophages and PBMCs of a pteropodid bat, *Pteropus alecto* showed dampened responses to LPS of *NLRP3*, an important component of the inflammasome, relative to humans and mice (13)*.* Similarly, a study of cell lines derived from big brown bats (*Eptesicus fuscus*) reported only weak up-regulation of *TNFα*, involved in systemic inflammation, following treatment with a dsRNA virus-associated PAMP, poly(I:C) (21). In the current study, we observed strong up-regulation of both *NLRP3* (fold-change = 43.5, P_adj._ = 2.71e-149) and *TNFα* (fold-change = 29.3, P_adj._ = 2.58e-141) in LPS-treated blood samples of *P. hastatus*. More generally, most genes with existing annotations for involvement in LPS response showed similar responses in bats as in other mammals; though there were exceptions among a few genes, including *IL23R*, *TNSFRSF11A* and *SLC11A1*. Importantly, LPS is a bacteria-associated molecular pattern, whereas much of the interest in bat immune defences and evidence for suppressed immune responses relates to viral infections (5, 10). While immune responses triggered by LPS will involve many of the same pathways as those triggered by other pathogens, our study may overlook adaptations directly implicated in dampened responses to viruses.

Crucially, we observed that immune responses showed considerable and predictable differences between individuals of the same species. Recent work has emphasised that bats are not a ‘monolith’, with substantial variation across taxa in pathogen exposure and immunity (28), as well as longevity (67) and other ecological and life history traits (7). Consistent with this, prior research has observed substantial variation across bats in the range and severity of physiological responses to LPS, including within the *Phyllostomidae* (68). Thus, while LPS triggers a strong proinflammatory transcriptomic response in *P. hastatus*, other—even closely related—bat species might differ in key aspects of this response. In the current study, we observed notable differences in immune response associated with sex and age, with short-lived males exhibiting a stronger pattern of immunosenescence. To what extent this pattern of male-biased immunosenescence contributes to, rather than simply reflects, sex-biased mortality represents an important direction for future research exploring the relationship between immunity and longevity. In this and other respects, our work adds to growing calls for studies of bats and their immune systems to consider both species and individual variation. Far from just representing noise to be statistically accounted for, comparisons among individuals of the same species provide powerful opportunities to identify factors that influence variation in immunological efficiency, with potential broader consequences for differences in aging and longevity.

## Materials and methods

### Sampling

Blood was collected from wild bats at two nearby locations in Trinidad (an abandoned ice storage building in Cumuto [10.5983°N, 61.2117°W] and a natural cave formation in Tamana [10.4711°N, 61.1958°W]), on three occasions: May 2023, January 2024, and January 2025. Groups of bats were captured from depressions in cave or building ceilings using a bucket with attached laundry hamper on the end of an extendable pole. Each bat was sexed and weighed to the nearest 0.5g, their forearm measured to the nearest 0.01mm, and tooth wear scored on a 10-point scale. The wing membrane of each wing was wiped with 70% isopropanol prior to collection of a 4mm diameter skin punch that was preserved in Zymo DNA/RNA Shield.

To collect blood, the vein running through the wing’s propatagium was pierced using a sterile lancet and a heparinized minivette (SARSTEDT product no. 17.2112.150) or capillary tube (Kimble) was used to collect 50ul of blood. Untreated blood samples were immediately preserved by mixing with 150ul of Zymo DNA/RNA Shield, while immune-stimulated samples were treated with 5μl of lipopolysaccharide (Invivogen, cat. no. *tlrl-peklps*) for a final concentration of 1μg/mL, and incubated at 38°C for three hours before preservation of RNA by addition of 150ul of Zymo RNA/DNA Shield. The order in which these samples were taken was alternated to avoid any confounding order effects. Untreated samples were immediately preserved due to unavoidable possibility of contamination with PAMPs under non-sterile field conditions, which could confound comparisons involving untreated samples.

Animal handling methods followed guidelines by the American Society of Mammalogists and were approved by the University of Maryland Institutional Animal Care and Use Committee under licences from the Forestry Division of the Ministry of Agriculture, Land and Fisheries, Trinidad and Tobago.

### Nucleic acid extraction, library preparation and sequencing

DNA and RNA were extracted from wing punches and blood samples using Zymo Quick-DNA Miniprep and Quick-RNA Whole Blood kits, respectively, following manufacturer’s protocols and including incubation with proteinase K (4 to 16 hours at 60C for DNA, 1 h at room temperature for RNA). DNA samples were shipped to the Clock Foundation for methylation profiling, as described in prior publications (49, 51). RNA Libraries were prepared using Illumina Stranded Total RNA with Ribo-Zero Plus using 350ng input of high integrity (RINe > 7) total RNA and 13 PCR cycles, with globin and ribosomal RNA sequences selectively removed using custom oligo pools. Whole-transcriptome sequencing was performed on an Illumina NextSeq 1000 platform to produce 25-35 million paired-end 50bp reads per library at the University of Maryland Brain and Behavior Institute’s Advanced Genomic Technologies Core. Adapter sequences were removed by the facility. We did not perform further trimming of reads. Gene expression was quantified by pseudoalignment of RNA sequences to the *Phyllostomus hastatus* genome (GCF_019186645.2) with Kallisto (v0.46.2) (69). We sequenced RNA from 70 untreated blood samples, and 96 LPS-treated samples. Sequencing was performed in 7 batches (independent sequencing runs), across 5 phases (independent batches of library preparation).

Small RNA libraries (N = 40) were prepared using RNA samples from 20 of the matched pairs of untreated and LPS-treated blood described above (10 of each sex) at the Institute for Genome Sciences, University of Maryland, Baltimore. Libraries were prepared from total RNA using Illumina miRNA prep kits and sequenced on an Illumina NovaSeq 6000 on an S4 flow cell to produce 10 million paired-end 100 bp reads per library. Reads were filtered using cutadapt (70) to retain those with length > 18 and < 26bp and subsequently processed using the miRDeep2 pipeline (71). Collapsed genome alignments were cross-referenced with miRNAs from humans, mice and three bat species (*Pteropus alecto*, *Eptesicus fuscus*, and *Artibeus jamaiciensis*) from miRbase (72), plus hairpin sequences of two more bat species (*Myotis myotis* and *Myotis lucifugus*) (73, 74). MiRDeep2 identified 2,128 putative novel miRNAs, which were filtered to retain 103 high-confidence miRNAs based on miRDeep2 assigned scores > 10, total read counts > 10, significant randfold P-values (indicating stable secondary structure), no existing miRBase miRNAs with the same seed, and no rfam alerts. We subsequently filtered out duplicate mature RNA and hairpin RNA sequences. Differential abundance analysis was performed in DESeq2. Overrepresentation of miRNA targets was tested using miRPath v4.0 (75), after performing the differential expression analysis only including human annotated miRNAs.

### Species comparison

Raw RNA-sequencing reads from baboons (39), macaques (38) and pigs (52) were retrieved from the corresponding sequencing repositories. Reads were aligned to respective transcriptome references from NCBI RefSeq (GCF_008728515.1, GCF_049350105.2, GCF_000003025.6) using Kallisto with 50 bootstraps and differential expression analysis was performed in DESeq2 using paired untreated or control, and LPS-treated blood samples from the same individuals. The pig dataset was collected from animals that were variable for a specific genotype, and we included only heterozygotes in our comparison. As the macaque sequencing data was in the form of single-end reads, we performed single-end transcript abundance estimated in Kallisto with two sets of parameters: fragment length 180 or 200bp, and standard deviation of 20 or 30, with the true values unknown. The different parameters produced near-identical differential expression results, so we used fragment length of 180bp and a standard deviation of 20 in our final analysis.

Genes encoding one-to-one protein orthologs were classified according to annotations from EggNog using the *emapper.py* script, with parameters: -m diamond, --evalue 0.001, --score 60, --pident 40, --query_cover 20, --subject_cover 20, -type proteins, –tax_scope 40674 (Mammalia), --target_orthologs one2one. Annotated orthologs were subsequently filtered to retain only those that were assigned to a single annotated gene in each species.

### Statistical analysis

Statistical analysis was performed in R v.4.4.2. Differential expression analysis was performed in DESeq2 (v1.44.0) (76). Batch effects were accounted for via inclusion as a fixed effect in models, except for paired analyses testing the effect of treatment between untreated and LPS-treated blood transcriptomes of the same animal. Differences associated with sampling season and year were confounded with sequencing batch, so were accounted for through its inclusion in models. Tests for differences in gene expression associated with sex and age each accounted for the other variable through its inclusion in the model (i.e., Expr ~ Sex + Age + Batch), except for single-sex models (Expr ~ Age + Batch). Tests of status effects in males accounted for age through its inclusion in the model (Expr ~ Age + Status + Batch). Initial exploratory visualisations through variance partitioning (77) suggested roost site (i.e., Tamana or Cumuto) had negligible effects on gene expression so it was not included in models. P-values were adjusted for multiple testing using a Benjamini-Hochberg correction with a false discovery rate of 0.05. No fold change criteria were imposed to consider a gene differentially expressed. Further linear mixed models, with batch effects incorporated throughout inclusion of random intercept, were run using lme4 and tested for significance using Wald’s Chi-squared with type II or type III (if an interaction was included) sums of squares.

Among LPS-treated samples, eleven out of 96 bats were sampled on more than one occasion, whereas among untreated bat samples, three bats were sampled on more than one occasion. In each case, bats were sampled a minimum of 8 months apart. This number of repeated samples was insufficient to account for individual ID in our models. We therefore included these repeated samples in our analyses but ensured the retention of just the first sample per bat did not influence general patterns of sex or age-related variation in immune profiles (Fig. S10-S12).

Gene ontology (GO) overrepresentation of biological processes was tested using Fisher’s exact tests implemented in ClusterProfiler (78), with a similarity threshold of 0.7 used to exclude overlapping GO terms. The overrepresentation of GO terms was tested against a background list of all genes included in the analysis that were assigned adjusted P-values. Gene ontology was assigned using human annotations, based on NCBI RefSeq gene symbols. KEGG and gene ontology overrepresentation of predicted miRNA targets was performed using miRPath 4.0 (75). Weighted gene co-expression network analysis was implemented in the R package WGCNA (79), with a minimum module size of 30 genes, and a merge cut height of 0.35. Prior to input into WGCNA, the sequencing batch effect was partitioned out of the normalized, variance stabilised counts using the ComBat function from the *sva* R package (80).

## Supplementary Material

Supplement 1
