# Supplementary material for: Interacting effects of sex and age on immune responses in a polygynous bat with male-biased mortality
Source: bioRxiv. 2026 Feb 27:2026.02.27.708485. Preprint. [Version 1] doi: 10.64898/2026.02.27.708485 (PMC13160085; doi:10.64898/2026.02.27.708485)
Supplement: Supplement 1 [file NIHPP2026.02.27.708485v1-supplement-1.pdf]

bioRxiv preprint doi: https://doi.org/10.64898/2026.02.27.708485; this version posted February 27, 2026. The copyright holder for this preprint (which was not certified by peer review) is the author/funder, who has granted bioRxiv a license to display the preprint in perpetuity. It is made available under aCC-BY 4.0 International license.

Supplemental Information

Little sex or age associated variation in miRNA abundance

No miRNAs were differentially expressed between males and females in untreated or LPS-treated blood samples. With respect to age, *pal-miR-599-3p* was significantly up-regulated in LPS-treated blood samples of older bats, while *hsa-miR-7-5p* was down-regulated. In each case, age-related patterns were consistent across sexes (Fig. S4). In humans, *miR-7-5p* is involved in suppressing pro-inflammatory NF-κB activity by decreasing expression of target genes, including *IL1β*, *IL6* and *IL8* (*81*), so its lower abundance in older bats is consistent with reduced suppression of inflammation. Leukocyte composition did not explain a substantial proportion of variation among miRNA profiles; NLR was significantly associated with the abundance of just one miRNA, *hsa-miR-4508*, and its inclusion as a covariate did not influence patterns of sex-biased or age-associated differences.

bioRxiv preprint doi: https://doi.org/10.64898/2026.02.27.708485; this version posted February 27, 2026. The copyright holder for this preprint (which was not certified by peer review) is the author/funder, who has granted bioRxiv a license to display the preprint in perpetuity. It is made available under aCC-BY 4.0 International license.

Supplementary figures

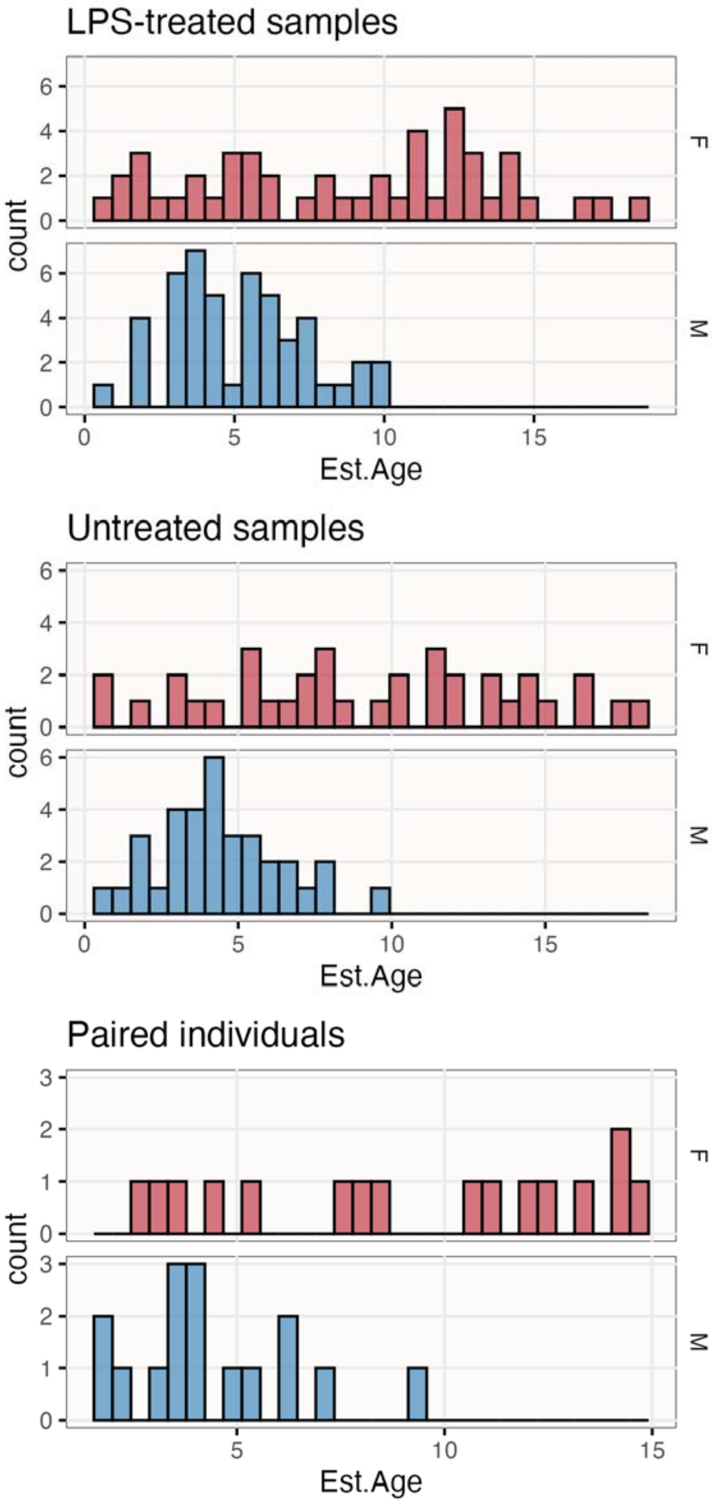

Figure S1. Distributions of samples across sexes and ages for untreated and LPS-treated whole-blood transcriptomes.

bioRxiv preprint doi: https://doi.org/10.64898/2026.02.27.708485; this version posted February 27, 2026. The copyright holder for this preprint (which was not certified by peer review) is the author/funder, who has granted bioRxiv a license to display the preprint in perpetuity. It is made available under aCC-BY 4.0 International license.

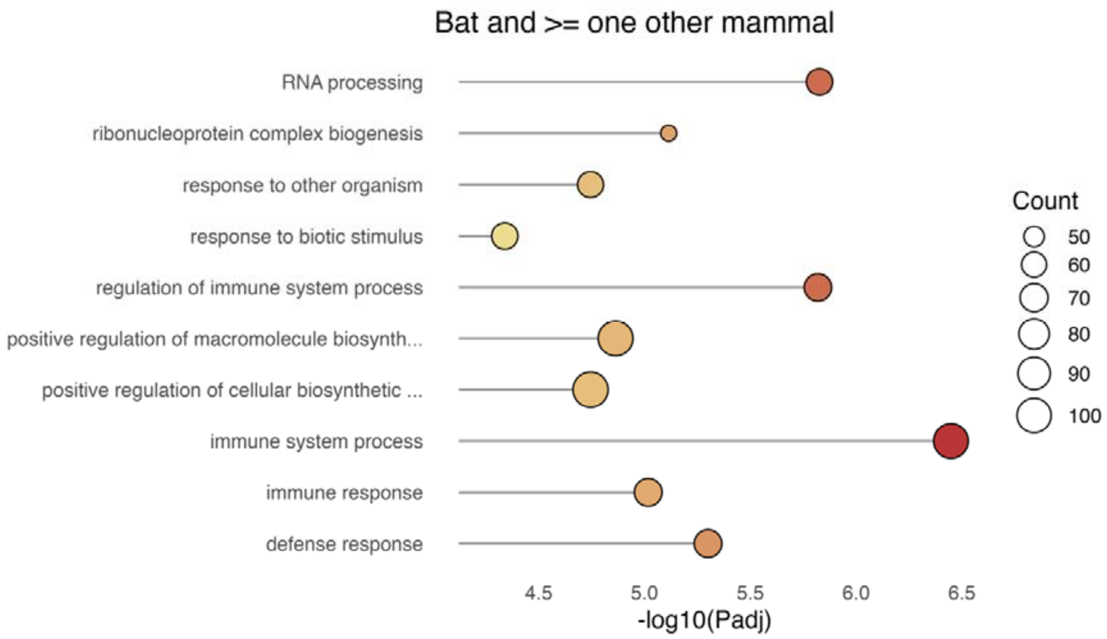

Figure S2. The top 10 overrepresented biological process gene ontology (GO) categories among genes that were up-regulated following LPS exposure in bats and at least one other mammal species.

bioRxiv preprint doi: https://doi.org/10.64898/2026.02.27.708485; this version posted February 27, 2026. The copyright holder for this preprint (which was not certified by peer review) is the author/funder, who has granted bioRxiv a license to display the preprint in perpetuity. It is made available under aCC-BY 4.0 International license.

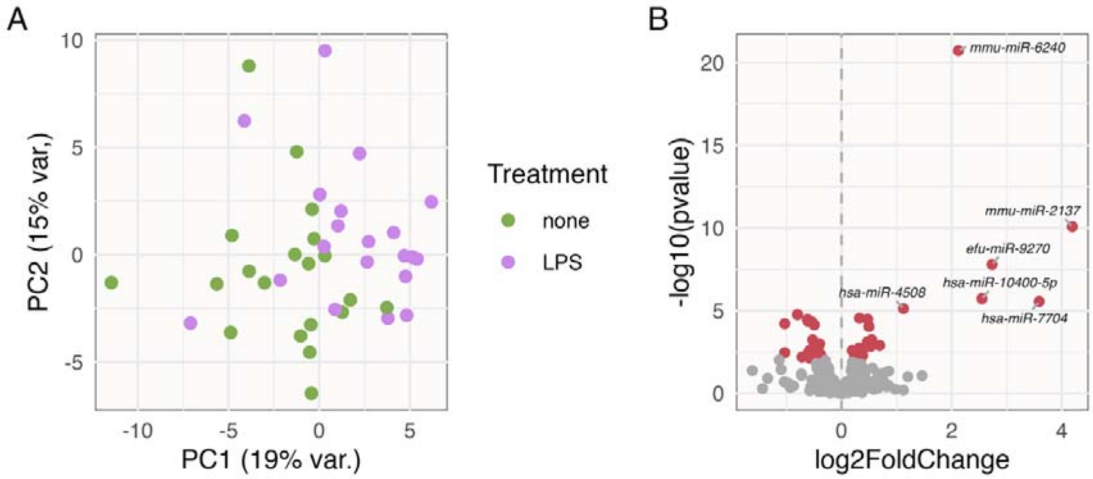

831

832 **Figure S3. Differential expression of miRNAs following LPS-treatment.**
833 **A)** Principal component analysis across miRNA transcripts from paired untreated and LPS-
834 treated samples. **B)** Differentially expressed miRNAs are highlighted in red.

835

bioRxiv preprint doi: https://doi.org/10.64898/2026.02.27.708485; this version posted February 27, 2026. The copyright holder for this preprint (which was not certified by peer review) is the author/funder, who has granted bioRxiv a license to display the preprint in perpetuity. It is made available under aCC-BY 4.0 International license.

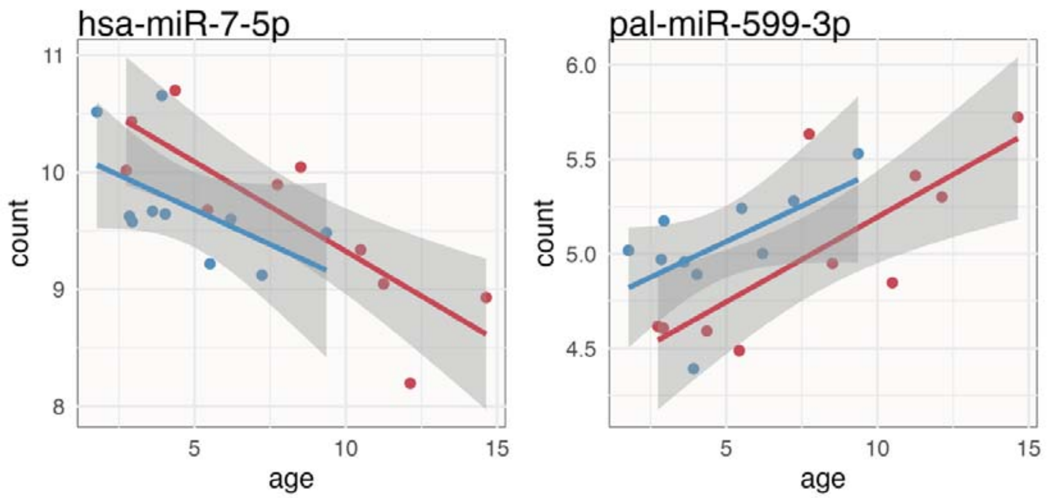

836

837 **Figure S4.** Patterns of age-associated variation in abundance of two miRNAs, showing
838 consistent patterns in males (blue) and females (red).

839

bioRxiv preprint doi: https://doi.org/10.64898/2026.02.27.708485; this version posted February 27, 2026. The copyright holder for this preprint (which was not certified by peer review) is the author/funder, who has granted bioRxiv a license to display the preprint in perpetuity. It is made available under aCC-BY 4.0 International license.

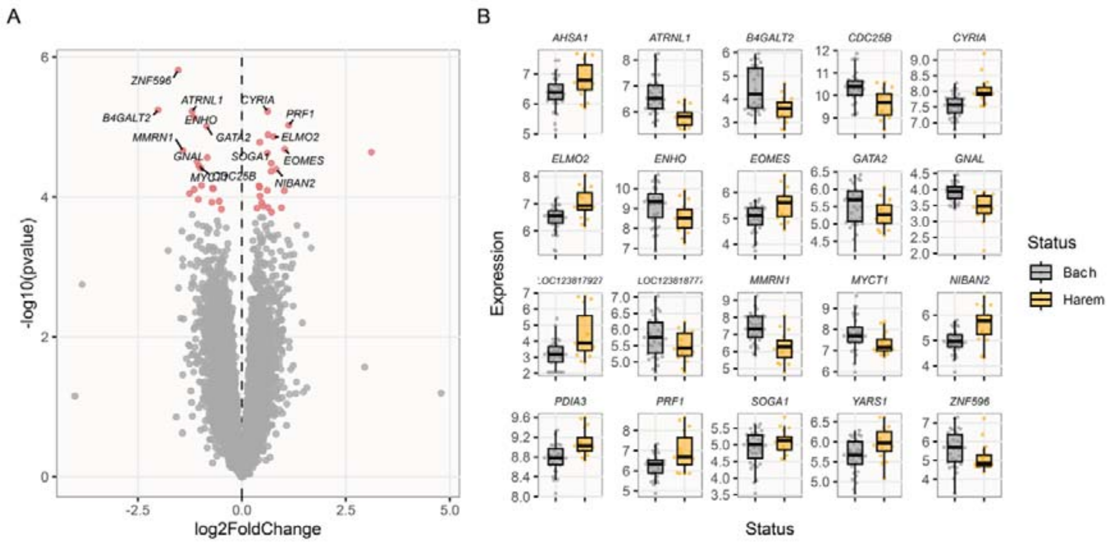

Figure S5. A) Volcano plot showing results of tests of differential expression between bachelor and harem males, using LPS-treated samples. Red points highlight genes differentially expressed at $P_{adj.} < 0.05$. B) Variance stabilised expression counts across bachelor (green) and harem (yellow) males, for the top 20 differentially expressed genes.

bioRxiv preprint doi: https://doi.org/10.64898/2026.02.27.708485; this version posted February 27, 2026. The copyright holder for this preprint (which was not certified by peer review) is the author/funder, who has granted bioRxiv a license to display the preprint in perpetuity. It is made available under aCC-BY 4.0 International license.

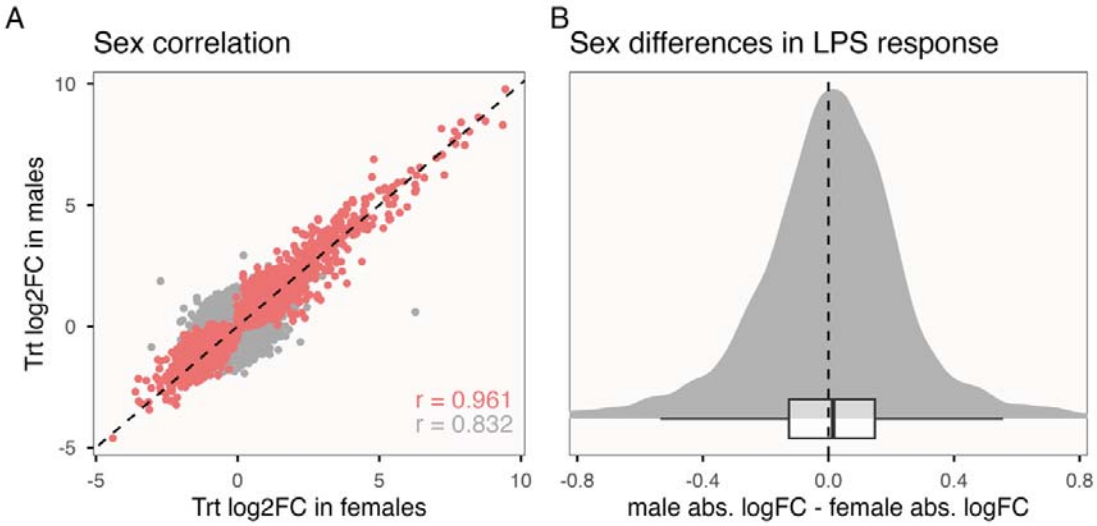

846

847 **Figure S6. Sex-specific responses to immune stimulus.**
848 **A)** Comparison of responses to LPS treatment in females and females, with significantly
849 LPS-affected genes highlighted in light red. Pearson's correlation coefficients are given for
850 significantly LPS-affected genes (red) and all genes (grey). **B)** Distribution of differences in
851 gene-wise log_2 fold-change across genes that responded in a consistent direction to LPS
852 stimulus in each sex.

bioRxiv preprint doi: https://doi.org/10.64898/2026.02.27.708485; this version posted February 27, 2026. The copyright holder for this preprint (which was not certified by peer review) is the author/funder, who has granted bioRxiv a license to display the preprint in perpetuity. It is made available under aCC-BY 4.0 International license.

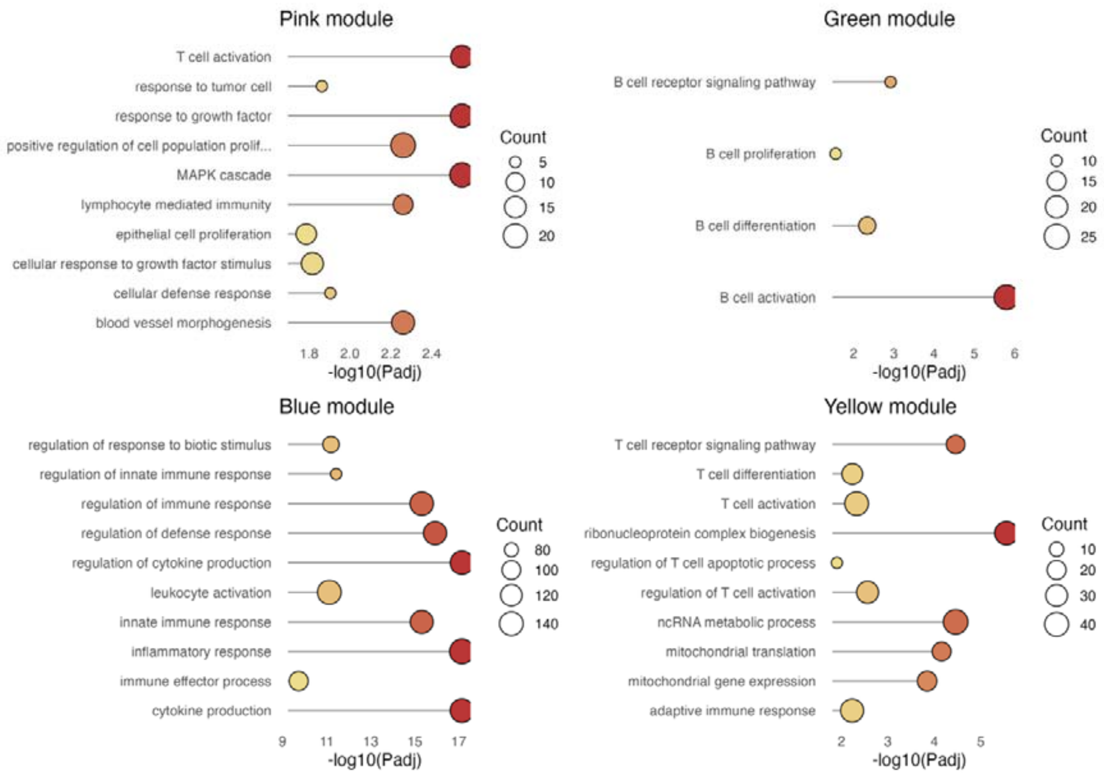

Figure S7. Overrepresented biological processes within each module of co-expressed genes found to be significantly associated with sex and/or age.

bioRxiv preprint doi: https://doi.org/10.64898/2026.02.27.708485; this version posted February 27, 2026. The copyright holder for this preprint (which was not certified by peer review) is the author/funder, who has granted bioRxiv a license to display the preprint in perpetuity. It is made available under aCC-BY 4.0 International license.

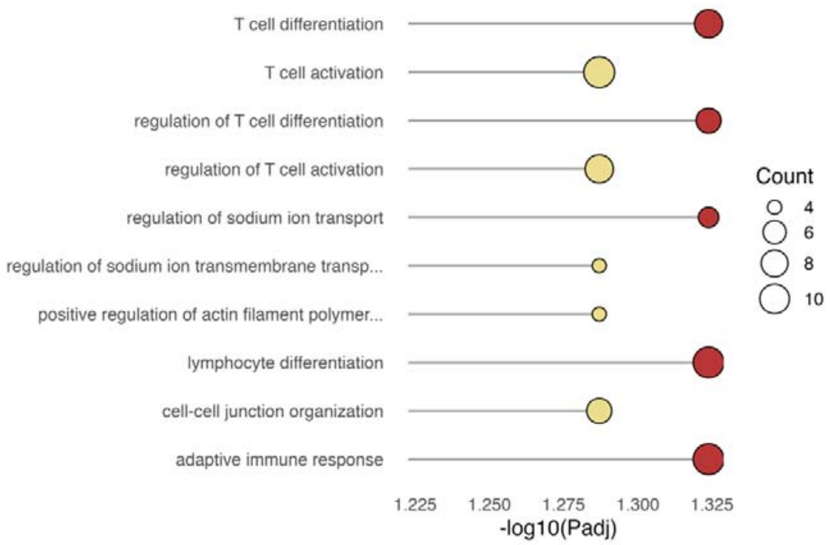

857

858 **Figure S8.** Overrepresented gene ontology categories among genes that were consistently
859 down-regulated with age in immune-stimulated transcriptomes of both sexes.

860

bioRxiv preprint doi: https://doi.org/10.64898/2026.02.27.708485; this version posted February 27, 2026. The copyright holder for this preprint (which was not certified by peer review) is the author/funder, who has granted bioRxiv a license to display the preprint in perpetuity. It is made available under aCC-BY 4.0 International license.

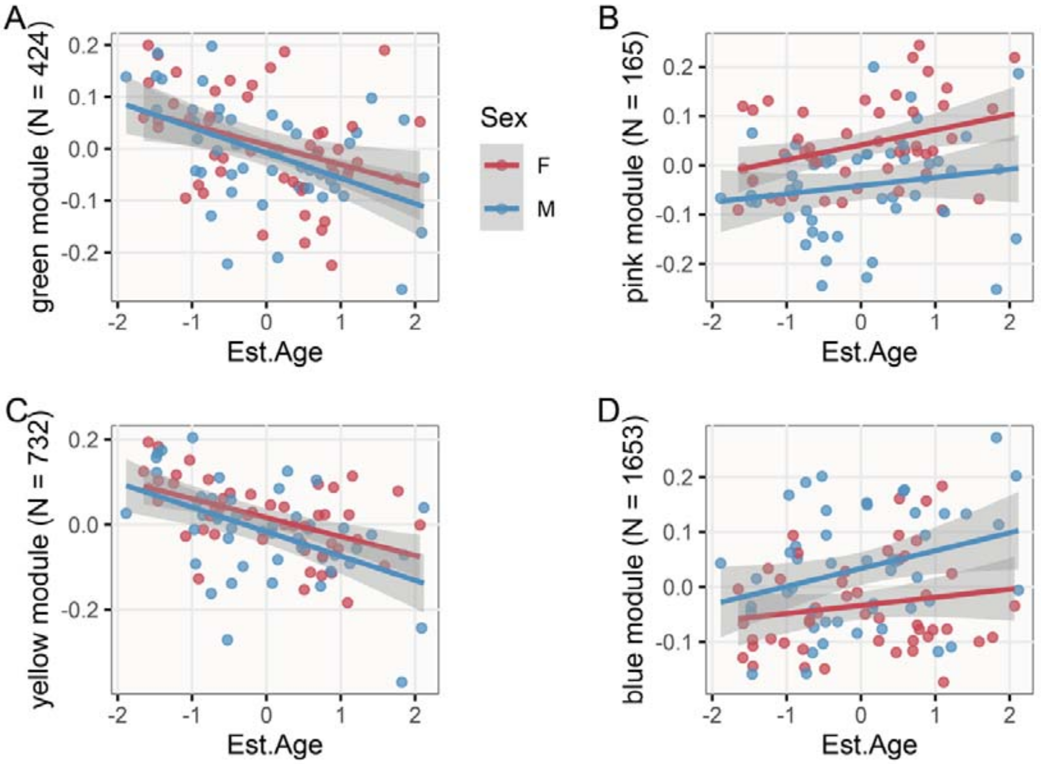

Figure S9. Patterns of expression for representative eigengenes from gene expression modules, corresponding to those in Fig. 2, after age is independently z-scaled for each sex. Lines represent results of sex-specific linear regression and 95% confidence intervals.

bioRxiv preprint doi: https://doi.org/10.64898/2026.02.27.708485; this version posted February 27, 2026. The copyright holder for this preprint (which was not certified by peer review) is the author/funder, who has granted bioRxiv a license to display the preprint in perpetuity. It is made available under aCC-BY 4.0 International license.

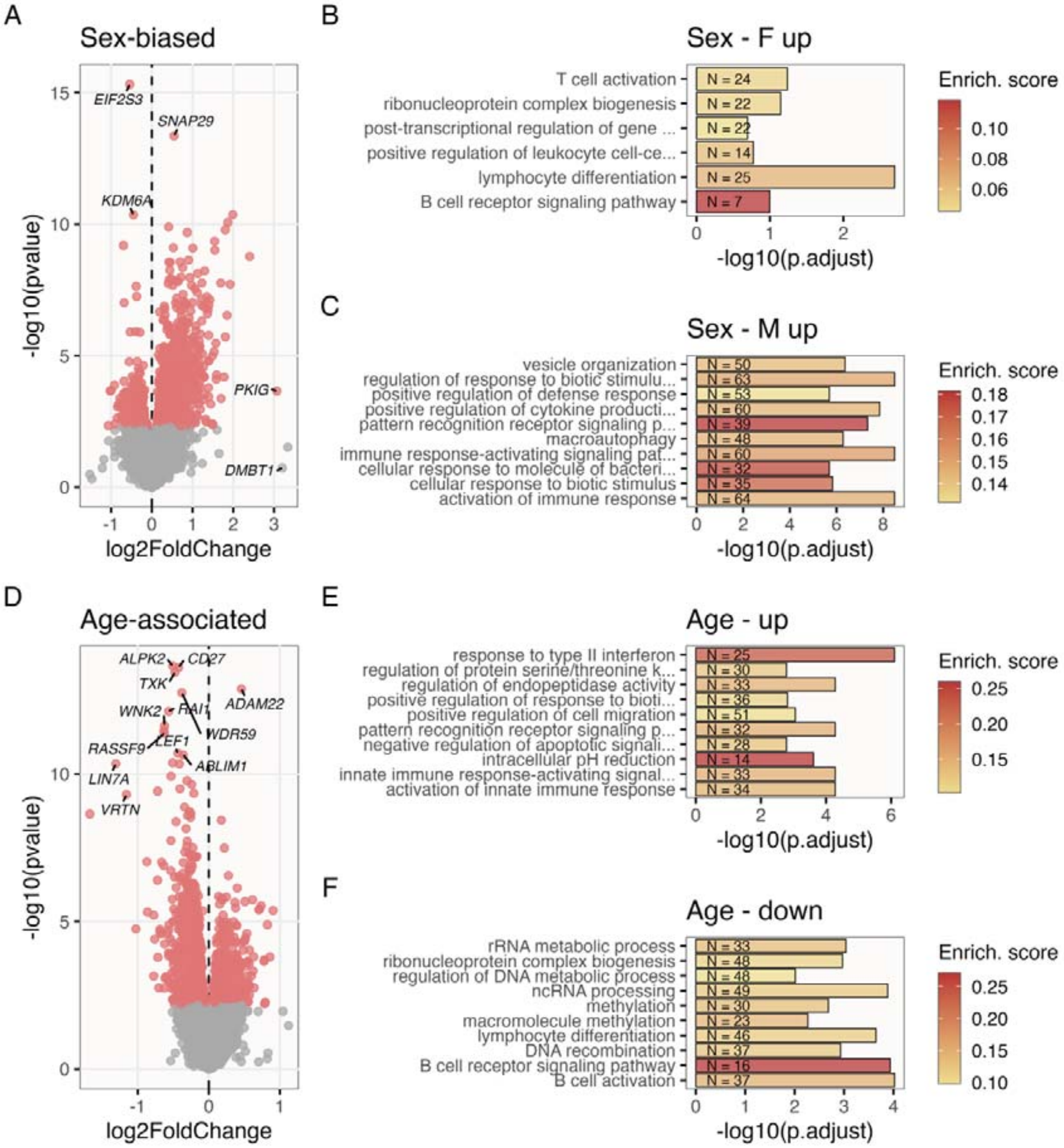

Figure S10. Sex and age-associated genes among LPS-treated transcriptomes, after repeated samples from the same individuals were removed.

bioRxiv preprint doi: https://doi.org/10.64898/2026.02.27.708485; this version posted February 27, 2026. The copyright holder for this preprint (which was not certified by peer review) is the author/funder, who has granted bioRxiv a license to display the preprint in perpetuity. It is made available under aCC-BY 4.0 International license.

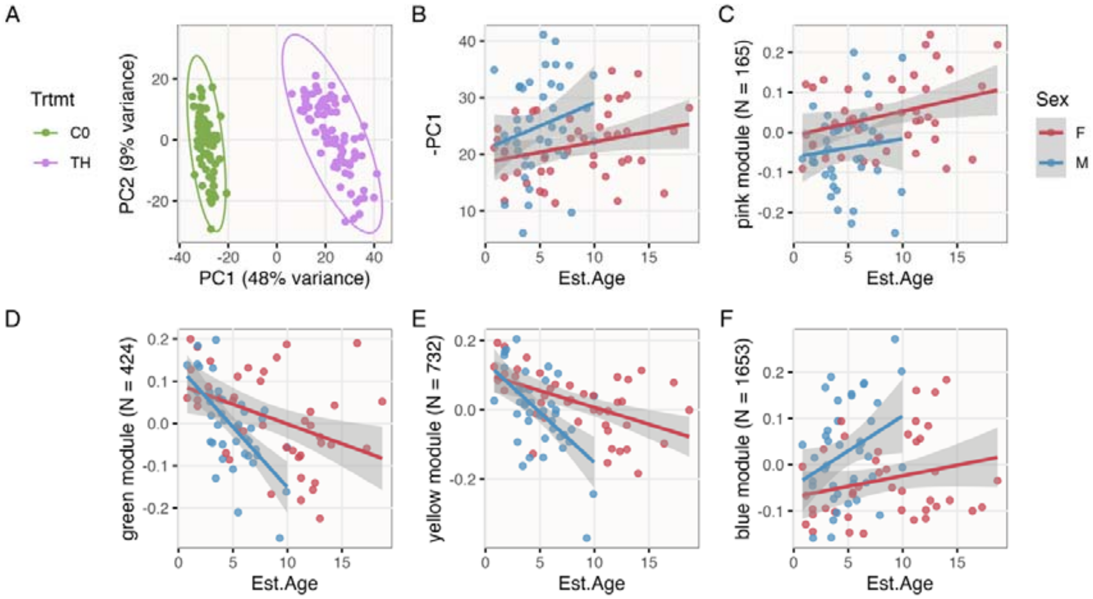

Figure S11. Patterns of sex and age-associated variation among LPS-treated transcriptomes (see Fig. 2), after repeated samples from the same individuals were removed.

bioRxiv preprint doi: https://doi.org/10.64898/2026.02.27.708485; this version posted February 27, 2026. The copyright holder for this preprint (which was not certified by peer review) is the author/funder, who has granted bioRxiv a license to display the preprint in perpetuity. It is made available under aCC-BY 4.0 International license.

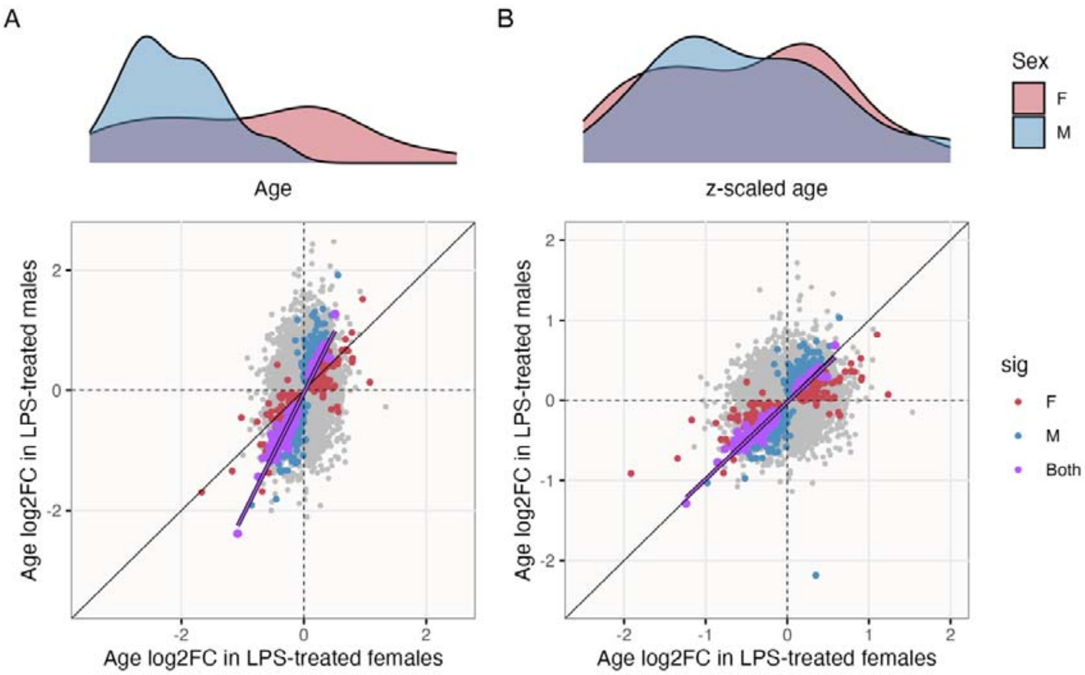

Figure S12. Sex differences in age-associated slopes across consistently age-associated genes among LPS-treated transcriptomes (see Fig. 3), after repeated samples from the same individuals were removed.
